# Betaglycan (TβRIII) is a Key Factor in TGF-β2 Signaling in Prepubertal Rat Sertoli Cells

**DOI:** 10.3390/ijms20246214

**Published:** 2019-12-09

**Authors:** Pradeep K Kudipudi, Sebastian P Galuska, Raimund Dietze, Georgios Scheiner-Bobis, Kate L Loveland, Lutz Konrad

**Affiliations:** 1 Center of Gynecology and Obstetrics, Faculty of Medicine, Justus Liebig University Giessen, 35392 Giessen, Germany; kpradeep2217@gmail.com (P.K.K.); raimund.dietze@imt.uni-marburg.de (R.D.); 2 Institute of Reproductive Biology, Leibniz Institute for Farm Animal Biology (FBN), 18196 Dummerstorf, Germany; galuska.Sebastian@fbn-dummerstorf.de; 3 Institute of Biochemistry, Faculty of Medicine, Justus Liebig University Giessen, 35392 Giessen, Germany; 4 Institute for Veterinary-Physiology and -Biochemistry, School of Veterinary Medicine, Justus-Liebig-University, 35392 Giessen, Germany; georgios.scheiner-bobis@vetmed.uni-giessen.de; 5 Centre for Reproductive Health, Hudson Institute for Medical Research, Melbourne 3168, Australia; kate.loveland@monash.edu; 6 Department of Molecular and Translational Sciences, School of Clinical Sciences, Monash University, Melbourne 3168, Australia

**Keywords:** Betaglycan, shedding, Smad, TGFbetas, TIMP3, MMPs, Sertoli cells

## Abstract

Transforming growth factor-βs (TGF-βs) signal after binding to the TGF-β receptors TβRI and TβRII. Recently, however, betaglycan (BG) was identified as an important co-receptor, especially for TGF-β2. Both proteins are involved in several testicular functions. Thus, we analyzed the importance of BG for TGF-β1/2 signaling in Sertoli cells with ELISAs, qRT-PCR, siRNA silencing and BrdU assays. TGF-β1 as well as TGF-β2 reduced shedding of membrane-bound BG (mBG), thus reducing the amount of soluble BG (sBG), which is often an antagonist to TGF-β signaling. Treatment of Sertoli cells with GM6001, a matrix metalloproteinases (MMP) inhibitor, also counteracted BG shedding, thus suggesting MMPs to be mainly involved in shedding. Interestingly, TGF-β2 but not TGF-β1 enhanced secretion of tissue inhibitor of metalloproteinases 3 (TIMP3), a potent inhibitor of MMPs. Furthermore, recombinant TIMP3 attenuated BG shedding. Co-stimulation with TIMP3 and TGF-β1 reduced phosphorylation of Smad3, while a combination of TIMP3/TGF-β2 increased it. Silencing of BG as well as TIMP3 reduced TGF-β2-induced phosphorylation of Smad2 and Smad3 significantly, once more highlighting the importance of BG for TGF-β2 signaling. In contrast, this effect was not observed with TIMP3/TGF-β1. Silencing of BG and TIMP3 decreased significantly Sertoli cell proliferation. Taken together, BG shedding serves a major role in TGF-β2 signaling in Sertoli cells.

## 1. Introduction

The TGF-β superfamily is a large group of proteins consisting of various TGF-β isoforms, bone morphogenetic proteins (BMPs), growth and differentiation factors, activins, inhibins, and glial-derived neurotrophic factors. It is well established that TGF-βs are crucial during testis development [1,2,3]. TGF-βs affect the proliferation and apoptosis of testicular germ and somatic cells [4,5]. TGF-β isoforms are highly expressed on mRNA level in Sertoli cells and their expression changes during development [6,7]. In spite of this, the quantities of active TGF-β proteins secreted by Sertoli cells into the medium are rather low [8,9]. In the developing testis, Sertoli cell proliferation and testis cord formation are regulated by TGF-β signaling [10]. In particular, TGF-β3 disturbs the blood–testis barrier possibly by targeting occludin, zonula occludens-1 and claudin-11 in junctional complexes [11]. 

TGF-βs signal through the type 1 (TβRI) and type 2 (TβRII) TGF-β receptors. Ligand binding to the extracellular domain of TGFβRII leads to TβRI kinase activation, which, in turn, phosphorylates receptor-activated Smads, Smad2 and Smad3 [12]. Phosphorylated Smad2/3 binds to Smad4 and the resulting complex can enter the nucleus regulating gene transcription with various co-activators and repressors [13,14,15]. TGF-βs also bind with high affinity to the co-receptors, betaglycan (TβRIII, BG) or endoglin [16], which may influence their function. In contrast to TGF-β1 and TGF-β3 which require only TβRI and TβRII to signal, TGF-β2 has a low affinity for TβRII, but binds with high affinity to betaglycan [17]: this binding mediates the interaction of TGF-β2 with TβRII and activates the signaling cascade for this ligand [18]. Thus, the abundance and localization of betaglycan is a key determinant of TGF-β pathway function and signaling outcomes. Betaglycan can further act as a receptor for inhibin to increase activin and BMP antagonism [19,20,21], or facilitate basic fibroblast growth factor (FGF-2) signaling by binding FGF-2 to its glycosaminoglycan chains [22,23]. 

Membrane-anchored betaglycan can be cleaved into a soluble form by matrix metalloproteinases (MMPs), a process known as shedding [24], thus sBG can act as an antagonist of TGF-β and BMP signaling [19,25,26]. Betaglycan shedding is mediated by the membrane-type MMPs, MT1-MMP, MT2-MMP and MT3-MMP [26]. The activity of the MMPs is inhibited by TIMPs which bind MMPs non-covalently in a 1:1 stoichiometry ratio [27,28]. Generally, there are 4 types of TIMPs (TIMP1–4) and they differ in their ability to block proteases [27]. However, TIMP3 is a major regulator of MMP activity in vivo [29]. 

TIMP1–3 are present in undifferentiated gonads (11.5 dpc) and neonatal testes [30]. Of note, TIMPs are themselves upregulated by TGF-βs [31,32,33] and they have complex regulatory interactions. TGF-βs induce TIMP3 synthesis via Smad3 and Smad4 [33].

In the normal testis betaglycan is expressed in somatic and germ cells in early stages of testis development including the fetus [34,35]. Its knockdown in mice results in fetal testis dysgenesis and compromised Leydig cell development [36], suggesting that betaglycan is essential for normal testis development and function.

Immature Sertoli cells proliferate until puberty, thus, regulation of Sertoli cell proliferation determines the final number. Cessation of proliferation is the prerequisite for Sertoli cell maturation and formation of the blood–testis barrier [37]. In the fetal testis blocking of TGF-β signaling significantly reduced Sertoli cell proliferation and prevents entry of male germ cells into mitotic arrest [10]. In this study we used an immortalized prepubertal Sertoli cell line to examine BG as a crucial regulator of TGF-β signaling and the modulating role of TIMP3. The results revealed clear differences in how TIMP3 and BG impact on pathway activation by TGF-β1 or TGF-β2 and effects on Sertoli cell proliferation.

## 2. Results

### 2.1. TGF-βs Increase mRNA Expression and Reduce Shedding of BG

**Figure 1:**
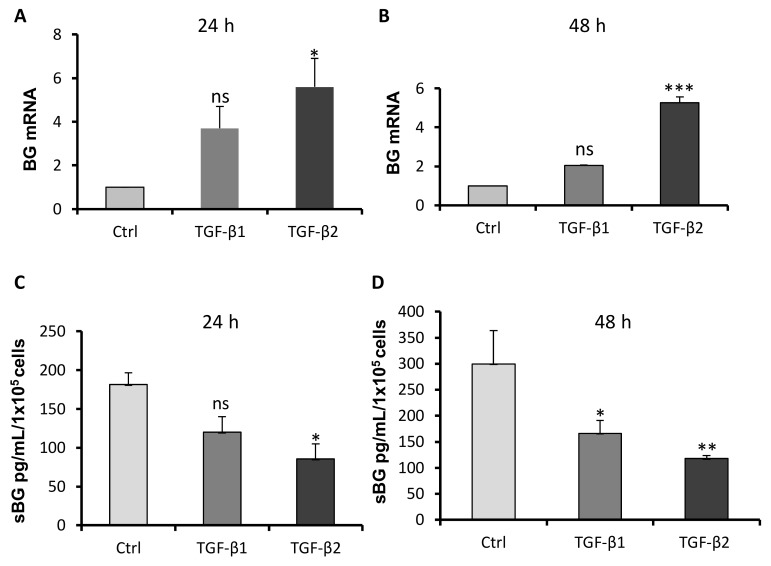
Effects of TGF-βs on mRNA expression and shedding of betaglycan (BG). The 93RS2 cells (7 × 10^4^ cells/well) were incubated with TGF-β1 or TGF-β2 (both 10 ng/mL) for 24 h and 48 h. mRNA expression was analyzed by qRT-PCR (**A**,**B**) and supernatants were analyzed by sBG ELISA (**C**,**D**). (**A**,**B**) TGF-β2 significantly increased mRNA expression of BG after 24 h and 48 h given as fold change compared to control. (**C**,**D**) TGF-β1 or TGF-β2 both decreased sBG values significantly after 48 h. Each bar represents the mean ± SEM of 3 independent experiments performed in duplicate. Dunnett’s test was used for statistical analysis; *p ≤ 0.05; **p < 0.01, ***p < 0.001 ns = not significant.

Since BG is an important co-receptor especially for TGF-β2 [38,39], we first sought to determine if TGF-βs modulate BG mRNA expression and shedding in Sertoli cells. For this purpose, we isolated mRNAs and supernatants from 93RS2 Sertoli cells treated with TGF-β1 or TGF-β2 for 24 h or 48 h. We found that TGF-β2, in particular, significantly increased mRNA expression of BG after 24 h as well as after 48 h (Figure 1A,B). Furthermore, both TGF-β1 as well as TGF-β2 reduced shedding of BG after 48 h significantly compared to untreated controls (Figure 1C,D). 

### 2.2. Effects of TGF-βs on TIMP3 Secretion and vice versa in SERTOLI Cells 

**Figure 2:**
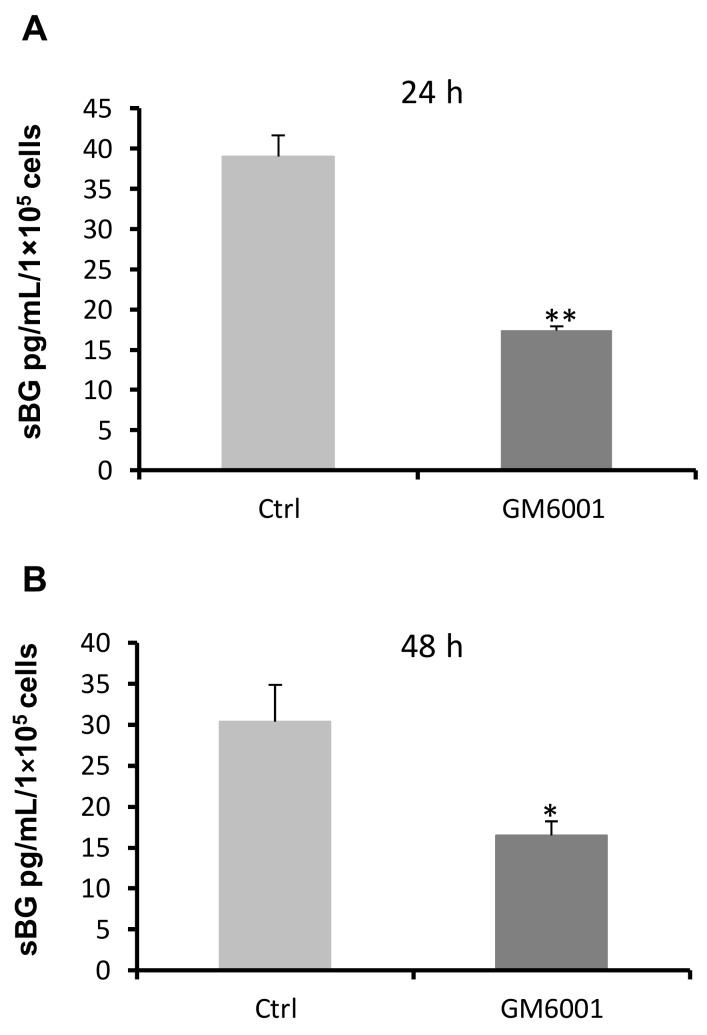
Matrix metalloproteinases (MMPs) regulate shedding of betaglycan. The 1 × 10^5^ 93RS2 cells/well were incubated with GM6001 (10 µM) for 24 h (**A**) and 48 h (**B**). Supernatants were analyzed for sBG by ELISA. GM6001 attenuated shedding of BG significantly. Each bar represents the mean ± SEM of 3 independent experiments performed in duplicate. Student’s *t*-test was used for statistical analysis; *p ≤ 0.05, **p < 0.01.

**Figure 3:**
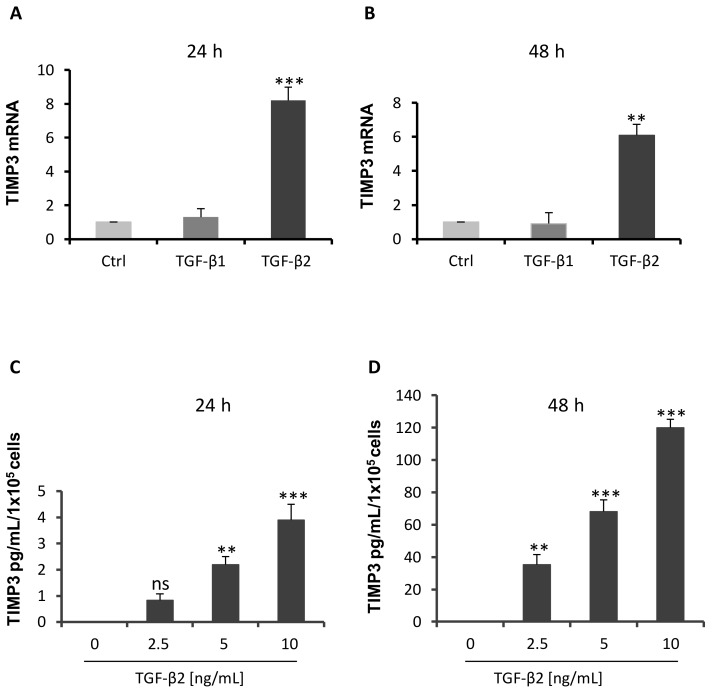
TGF-β2 treatment induces TIMP3 mRNA and secretion. The 1 × 10^5^ 93RS2 cells/well were incubated with TGF-β1 or TGF-β2 (both 10 ng/mL) for (**A**) 24 h or (**B**) 48 h and the mRNA expression of TIMP3 measured with qRT-PCR. Only TGF-β2 stimulated TIMP3 mRNA expression significantly given as fold change of control. 1 × 10^5^ 93RS2 cells/well were incubated with TGF-β2 for (**C**) 24 h or (**D**) 48 h. Supernatants were analyzed for TIMP3 by ELISA. TGF-β2 stimulated secretion of TIMP3 dose-dependently and significantly. Each bar represents the mean ± SEM of 3 independent experiments performed in duplicate. Dunnett’s test was used for statistical analysis; **p < 0.01, ***p < 0.001, ns = not significant.

MMPs and TIMPs regulate shedding of BG in rat muscle cells [36]. Investigation of the impact of MMPs on BG shedding using the broad range MMP inhibitor GM6001 demonstrated reduced sBG values by about ~50% after 24 h and 48 h (Figure 2). In vivo, TIMP1–3 are the major inhibitors of MMPs, thus, we analyzed secretion of TIMPs in 93RS2 Sertoli cells cultured with or without TGF-βs. Because neither TIMP1 nor TIMP2 (62.5 pg/mL detection limit) could be detected in 48 h culture medium or after stimulation with different doses of TGF-β1 or TGF-β2, we focused on TIMP3. Only TGF-β2 but not TGF-β1 induced TIMP3 mRNA expression significantly (Figure 3A,B). Similarly, TGF-β2 (Figure 3C,D), but not TGF-β1, induced secretion of TIMP3 in a dose-dependent and significant manner. The 48 h samples contained about ~30 times more TIMP3 than the 24 h samples.

### 2.3. Effects of TIMP3 on TGF-βs and on Shedding of BG 

**Figure 4:**
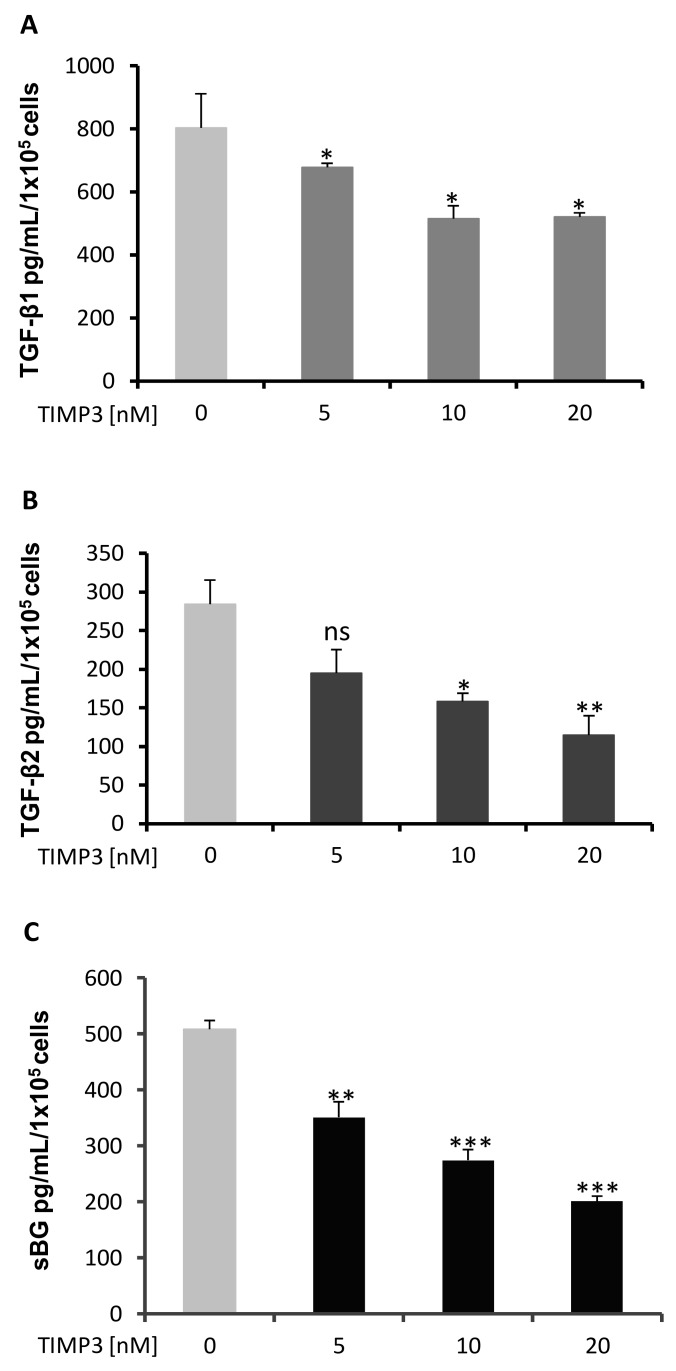
TIMP3 treatment reduces secretion of TGF-β1, TGF-β2 and shedding of BG. The 1 × 10^5^ 93RS2 cells/well were incubated with TIMP3 for 48 h. Supernatants were analyzed for TGF-β1 (**A**), TGF-β2 (**B**) and sBG (**C**) by ELISA. TIMP3 reduced secretion of TGF-β1 (**A**), TGF-β2 (**B**) and shedding of sBG (**C**) dose-dependently and significantly. Each bar represents the mean ± SEM of 3 independent experiments performed in duplicate. Dunnett’s test was used for statistical analysis; *p ≤ 0.05, **p < 0.01, ***p < 0.001, ns = not significant, rhTIMP3 = recombinant TIMP3, ns = not significant.

Next, we analyzed the effects of TIMP3 on secretion of TGF-βs and BG shedding. 93RS2 Sertoli cells were treated with different doses of TIMP3 for 48 h and the contents of TGF-βs and sBG determined. Both TGF-β1 (~800 pg/mL/1 × 10^5^ cells) and TGF-β2 (~300 pg/mL/1 × 10^5^ cells) were detected in 48 h culture supernatants from 93RS2 cells (Figure 4A,B). Treatment with TIMP3 caused a dose-dependent and significant decrease in secretion of TGF-β1 (~40% reduction with 10 nM and 20 nM of TIMP3) and of TGF-β2 (~70% reduction with 20 nM TIMP3). Similarly, the concentration of sBG was reduced in a dose-dependent and significant manner by up to ~60% with 20 nM TIMP3 (Figure 4C). Treatment of Sertoli cells with TIMP3 was without any effects on cell viability (Figure S1). 

### 2.4. The Roles of TIMP3 and BG in TGF-β Signaling 

**Figure 5:**
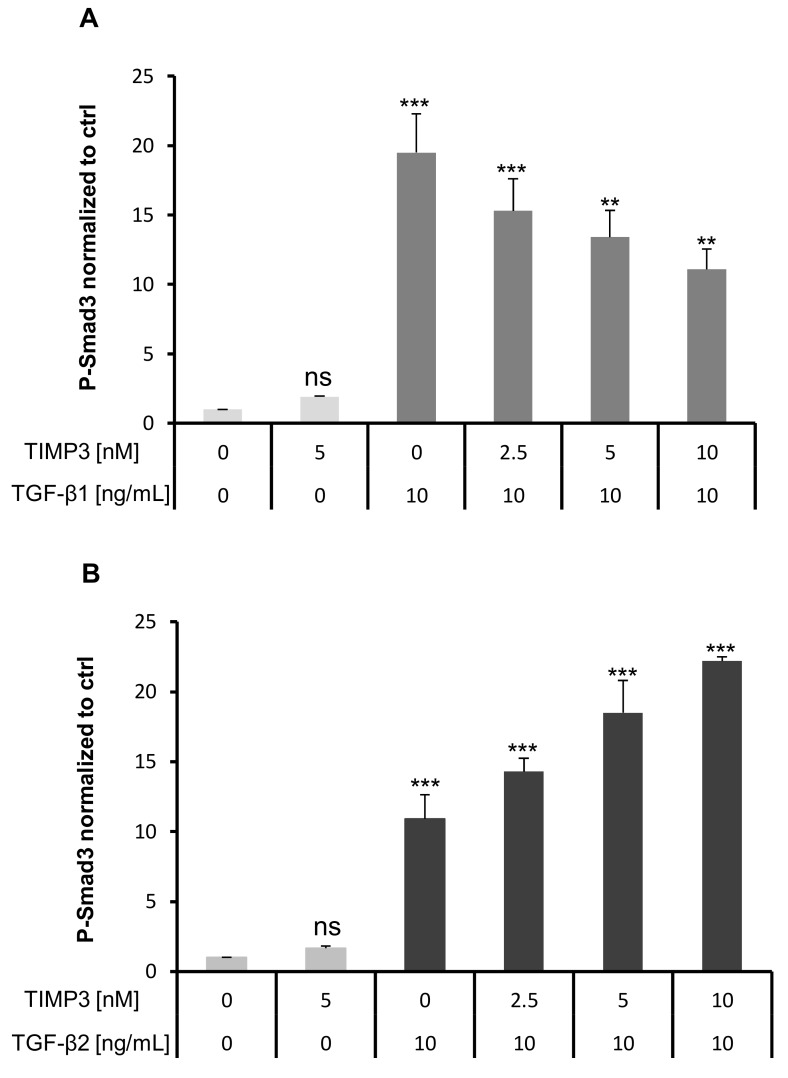
Effects of TIMP3 on Smad3 phosphorylation in TGF-β1/2 signaling. The 7 × 10^4^ 93RS2 cells/well were incubated with different doses of TIMP3 for 2 h and then treated with (**A**) TGF-β1 or (**B**) TGF-β2 (both 10 ng/mL) for 2 h. Cell lysates were analyzed for p-Smad3 by ELISA. TIMP3 only increased phosphorylation of Smad3 together with TGF-β2 (**B**) dose-dependently and significantly. Each bar represents the mean ± SEM of 3 independent experiments performed in duplicate. Dunnett’s test was used for statistical analysis; **p < 0.01; ***p < 0.001, ns = not significant.

**Figure 6:**
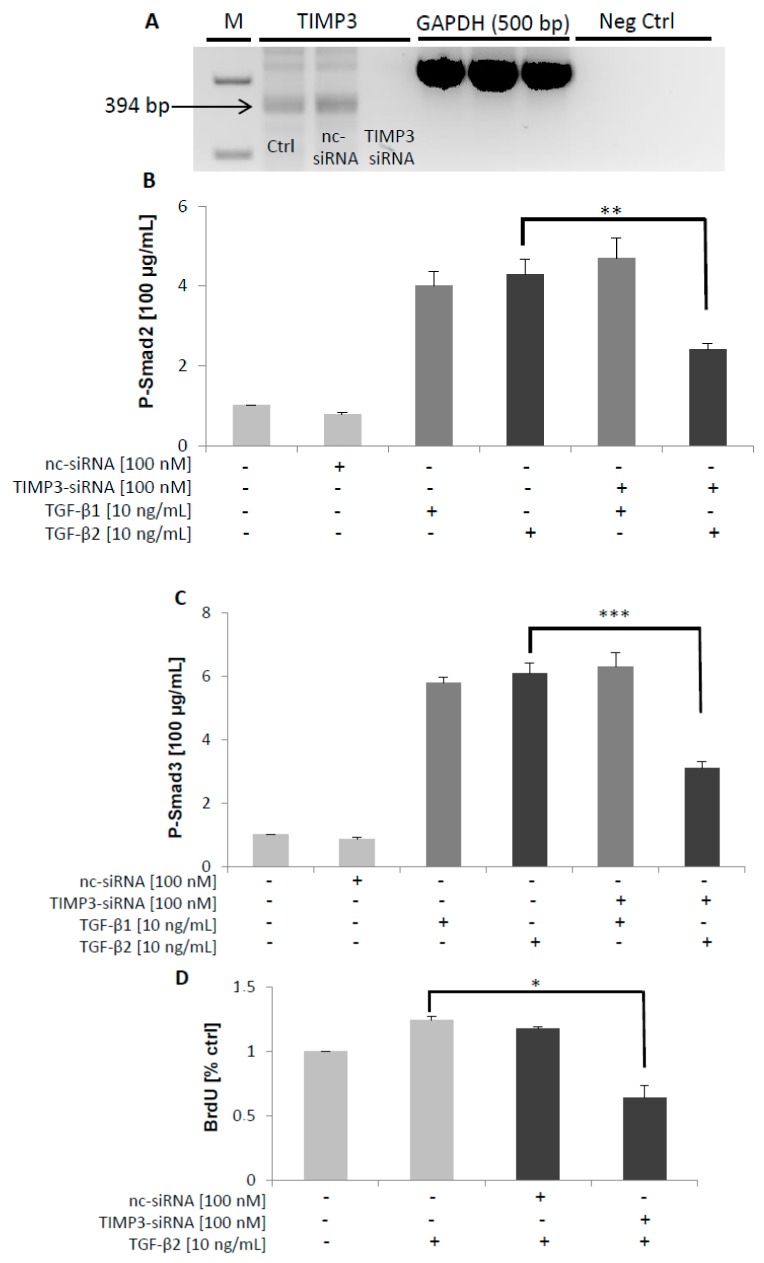
Effects of TIMP3 silencing on Smad2/3 phosphorylation in TGF-β1/2 signaling. A control RT-PCR of the knockdown effectiveness for TIMP3 was performed to ensure silencing of TIMP3 (**A**). The 7 × 10^4^ 93RS2 TIMP3-silenced cells were treated with TGF-β1 or TGF-β2 (both 10 ng/mL) for 1 h. Cell lysates were analyzed for P-Smad2 and P-Smad3 by ELISAs. Silencing of TIMP3 attenuated TGF-β2 but not TGF-β1-dependent phosphorylation of Smad2 (**B**) and of Smad3 (**C**) as well as proliferation of 93RS2 cells by 50% (**D**). Each bar represents the mean ± SEM of 3 independent experiments performed in duplicate. Dunnett’s test was used for statistical analysis; *p ≤ 0.05, **p < 0.01; ***p < 0.001; Ctrl, control; nc-siRNA, negative control siRNA; M, Marker; +, addition of substance; -, without addition of substance.

**Figure 7:**
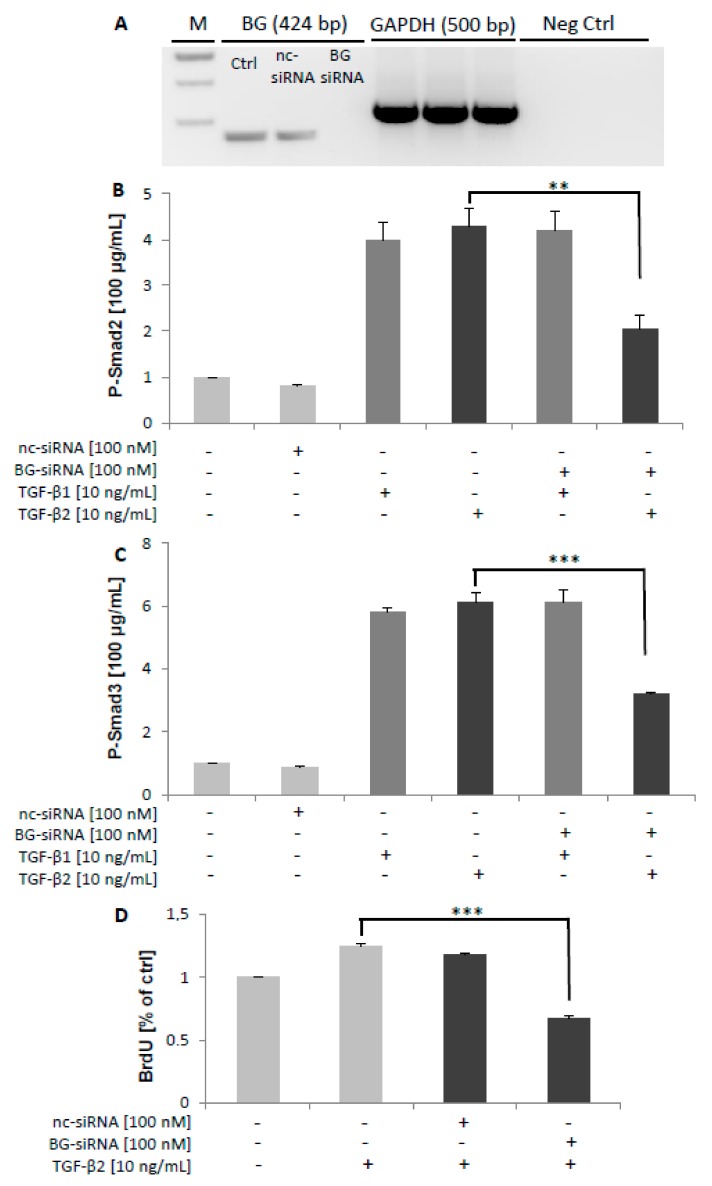
Effects of BG silencing on Smad2/3 phosphorylation in TGF-β1/2 signaling. A control RT-PCR of the knockdown effectiveness for BG was performed to ensure silencing of BG (**A**). The 7 × 10^4^ 93RS2 BG-silenced cells were treated with TGF-β1 or TGF-β2 (both 10 ng/mL) for 1 h. Cell lysates were analyzed for P-Smad2 and P-Smad3 by ELISAs. Silencing of BG attenuated TGF-β2 but not TGF-β1-dependent phosphorylation of Smad2 (**B**) and of Smad3 (**C**) as well as proliferation of 93RS2 cells by 47% (**D**). Each bar represents the mean ± SEM of 3 independent experiments performed in duplicate. Dunnett’s test was used for statistical analysis; **p < 0.01; ***p < 0.001; Ctrl, control; nc-siRNA, negative control siRNA; M, Marker; +, addition of substance; -, without addition of substance.

It has been reported that mBG inhibited TGF-β signaling by interfering with TβRI and TβRII [40]. Because TIMP3 treatment reduced BG shedding as shown above, we wanted to elucidate the effects of increased mBG on TGF-β signaling. Thus, we pre-treated Sertoli cells with TIMP3 for 2 h and then stimulated with TGF-β1 or TGF-β2 for 2 h. The time point of 2 h for the P-Smad3 ELISA was optimized before the stimulation was done. Of note, we observed that titrated TIMP3 reduced TGF-β1-dependent phosphorylated Smad3 levels at 2 h (Figure 5A), whereas the converse occurred for TGF-β2 (Figure 5B). These results support the hypothesis that BG is substantially required for TGF-β2 but not for TGF-β1-mediated signaling. This assumption was further corroborated by the observation that silencing of TIMP3 reduced TGF-β2-induced but not TGF-β1-induced phosphorylation of Smad2 and Smad3 (Figure 6B,C) resulting in reduced proliferation of 93RS2 Sertoli cells (Figure 6D). Furthermore, silencing of BG reduced TGF-β2-induced but not TGF-β1-induced phosphorylation of Smad2 and Smad3 (Figure 7B,C) also resulting in reduced proliferation of 93RS2 Sertoli cells (Figure 7D).

## 3. Discussion

Though functionally diverse, all human TGF-beta family ligands signal through a limited number of receptors [41]. Especially for TGF-β1, -β2 and -β3 signaling via TβRI and TβRII was described. However, this could not account for the functional diversity such as the importance of TGF-β1 in inflammation [42], the impact of TGF-β2 in several developmental defects [43] and TGF-β3-dependent deficiencies in lung and cleft palate [44] observed in vivo by silencing of the TGF-βs. In contrast to TGF-β1 and TGF-β3, which do not need betaglycan to signal, TGF-β2 has only a weak affinity for TβRII and thus requires betaglycan for signaling [45]. It was recently shown that betaglycan binds TGF-β2 with a 1:1 stoichiometry and concentrates TGF-β2 on the cell surface for binding to TβRII [46]. Thereafter, betaglycan is replaced by TβRI in the receptor complex for signaling. In this study, we have elaborated on the differences of TGF-β1 and TGF-β2 signaling and impact on proliferation using immortalized Sertoli cells as a model system.

In a first set of experiments, we analyzed whether TGF-βs influenced betaglycan and found an increased mRNA expression of betaglycan, especially by TGF-β2. Furthermore, we could show in accordance with the literature [47] that ligand binding inhibited betaglycan shedding. Since MMPs and especially MT1-MMP were described to be involved in betaglycan shedding [24], we first used the general MMP inhibitor GM6001 and found reduced BG shedding. Because in vivo, the TIMPs are the main MMP inhibitors, we also analyzed the influence of TIMPs. 

Interestingly, we found that only TGF-β2, and not TGF-β1, induced mRNA expression and secretion of TIMP3, whereas TIMP1 and TIMP2 were not found to be secreted by 93RS2 Sertoli cells regardless of treatment with TGF-βs. Interestingly in vivo TIMP3 is the main MMP inhibitor because it is present in nearly all tissues and can inhibit all MMPs [27,31,48]. Furthermore, we observed that TIMP3 counteracted TGF-β1 and TGF-β2 secretion and perhaps more importantly reduced betaglycan shedding. Although these results indicated that TIMP3 is an important regulator in Sertoli cells to reduce shedding of betaglycan it was not clear whether this effect might positively influence TGF-β signaling.

It has been reported that membrane-bound betaglycan can inhibit Smad-dependent TGF-β signaling by interfering with TβRI and TβRII after stimulation with TGF-β1 [40,49]. Because we have shown that TIMP3-reduced shedding of betaglycan resulted in increased Smad3 phosphorylation, we wanted to analyze whether TGF-β1 or TGF-β2 are also involved. Thus, we pre-treated Sertoli cells with TIMP3 and then stimulated them with TGF-β1 or TGF-β2. We observed that TIMP3 together with TGF-β1 strongly reduced P-Smad3 levels consistent with published results [40,49]. Remarkably, however, phosphorylation of Smad3 was increased when Sertoli cells were treated with TIMP3 together with TGF-β2. These results strongly suggest that betaglycan is required for TGF-β2, but not for TGF-β1, signaling.

In a last setup of experiments, we analyzed the effects of TIMP3 and betaglycan silencing on TGF-β signaling and the cellular consequences. We could clearly show that silencing of TIMP3 as well as of betaglycan strongly attenuated TGF-β2-dependent but not TGF-β1-dependent phosphorylation of Smad2 and of Smad3. Silencing of TIMP3 as well as of betaglycan attenuated cell proliferation of 93RS2 Sertoli cells which is in accordance to significant decreases in the percentage of proliferating cells in betaglycan^-/-^ embryos [50]. Our results further corroborate our assumption that betaglycan and TIMP3 are pivotal for TGF-β2, but not for TGF-β1 signaling.

One of the limitations of the current study is the use of immortalized prepubertal Sertoli cells. Although these cell lines do not possess all biological functions of primary Sertoli cells [51,52], the impurity of primary Sertoli cells is a major concern for studying signaling as in the current study. Contamination of Sertoli cells by peritubular cells, which are also positive for TGF-β receptors, is also a problem. Furthermore, peritubular cells would have been a serious confounder especially in our study, because they secrete TGF-βs, which affect Sertoli cell functions [53]. In addition to follicle-stimulating hormone (FSH), the insulin family of growth factors, activin, several cytokines [37] and TβRI [10], we clearly demonstrated the involvement of betaglycan and TIMP3 in proliferation of prepubertal Sertoli cells comparable to in vivo results [10,37]. A further limitation of the current study is the use of ELISAs for detection of phosphorylated Smad2 and Smad3. A Western blot would have been better suited to discriminate between Smad2 and Smad3, however, even with some cross-reactions in detection of P-Smad2 and P-Smad3, we have clearly shown that betaglycan as well as TIMP3 only influence Smad-dependent signaling of TGF-β2 and definitely not of TGF-β1. 

The next steps to be done include the elucidation of the contribution of the MMPs into shedding of betaglycan. Furthermore, our new data concerning the importance of betaglycan and TIMP3 for TGF-β2 signaling should be elaborated with more cell lines or primary cells. Indeed, first preliminary experiments with human endometrial cell lines have shown similar results. Equally important are experiments with cell-specific betaglycan knockouts, especially in Sertoli cells, in order to obtain in vivo results.

**Figure 8:**
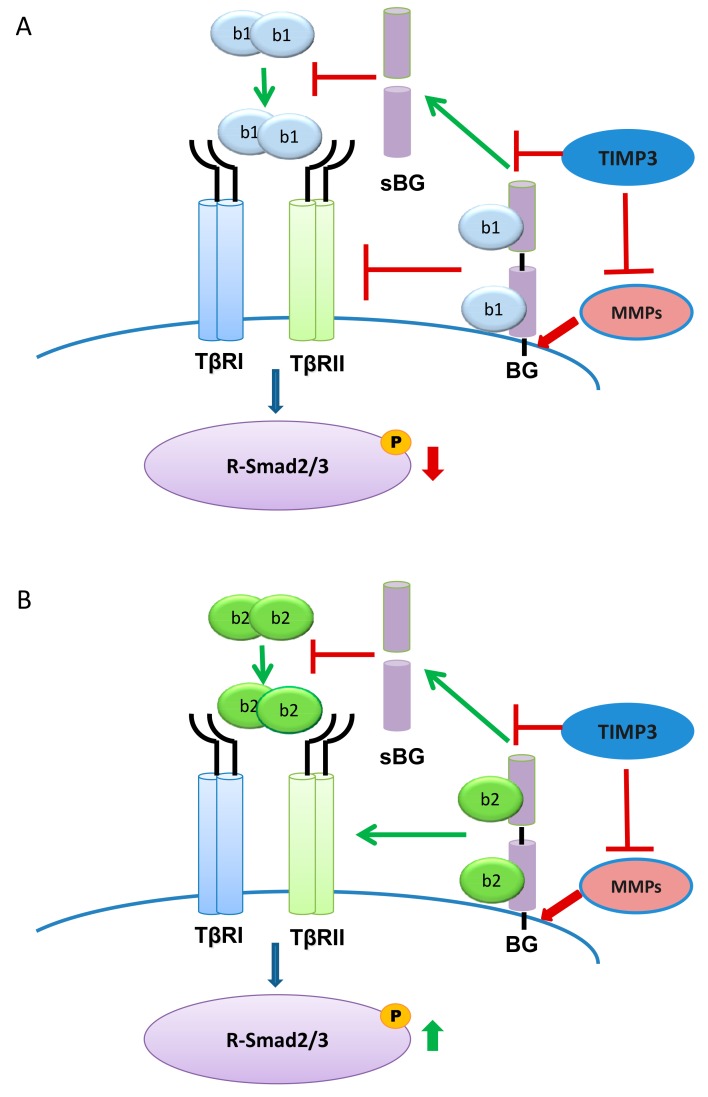
Scheme for the different modes of signaling by TGF-β1 and TGF-β2 under the influence of BG and TIMP3. (**A**) Normally binding of TGF-β1 (b1) to the TGF-β receptor complex results in phosphorylation of Smad3. However, TIMP3 counteracts MMP-mediated BG shedding, thus resulting in interference of membrane-bound and ligand-activated BG with the TGF-β receptor complex. This interaction attenuates TGF-β1-dependent Smad3 phosphorylation via TβRI. sBG reduces binding of TGF-β1 to the TGF-β receptor complex and thus counteracts TGF-β1 signaling. (**B**) In contrast, binding of TGF-β2 (b2) to BG enhances phosphorylation of TGF-β2-dependent Smad3 which is further increased when TIMP3 inhibits MMP-dependent BG shedding. sBG reduces binding of TGF-β2 to the TGF-β receptor complex and thus counteracts TGF-β2 signaling.

Taken together, we found that betaglycan is an important co-receptor for TGF-β signaling, especially for TGF-β2 (Figure 8). In addition to modulation of betaglycan shedding by the ligands, TIMP3 is an important regulator of this process. Silencing of TIMP3 and betaglycan underscored the importance of both proteins in signaling of TGF-β2 and in cell proliferation of Sertoli cells. Thus, we have clearly demonstrated the substantial need for betaglycan in TGF-β2 signaling (Figure 8).

## 4. Materials and Methods

### 4.1. Cell Culture

The prepubertal rat Sertoli cell line 93RS2 [54] was provided by Dr. Boekelheide and has been intensively characterized [52]. We use secretion of clusterin as a highly specific Sertoli cell marker and SOX9 staining in cell culture for purity of the cell line. The 1 × 10^5^ cells were maintained in DMEM/Ham’s F-12 supplemented with 2 mM l-glutamine, 1% streptomycin/penicillin, 10% fetal calf serum (FCS) and 1% insulin transferrin-selenium and cultured in a humidified incubator (37 °C, 5% CO_2_). All cell culture reagents were obtained from Life Technologies (Darmstadt, Germany). Medium was replaced every 2 days and cells were routinely passaged after reaching 70–80% confluency. For harvest the medium was aspirated and after a washing step with 1× PBS without Ca^2+^ and Mg^2+^ cells were detached by incubation with accutase (0.25%) for 4 min at 37 °C. Then cells were either stored or resuspended at 1 × 10^6^ cells/mL for experimental use. 

### 4.2. Recombinant Proteins, Inhibitors and ELISA Kits

We used the following materials and kits: recombinant human TIMP3 (R&D Systems, Wiesbaden, Germany) and recombinant human TGF-β1 and TGF-β2 (Promokine, Heidelberg Germany), protease inhibitor cocktail, Fluoroprofile protein quantification kit (Merck/Sigma Aldrich, Darmstadt, Germany), siRNAs (Thermo Fisher/Invitrogen, Carlsbad, CA, USA) and GM6001 (broad-spectrum inhibitor of MMPs; Sigma Aldrich, St. Louis, Missouri, USA), TGF-β1 (range 31.2–2000 pg/mL, sensitivity 15.6 pg/mL, cat-no MB100B), TGF-β2 (range 31.2–2000 pg/mL, sensitivity 15.6 pg/mL, cat-no DY302), and TIMP3 (range 62.5–4000 pg/mL, cat-no DY973) ELISAs (all from R&D Systems, Wiesbaden, Germany), TIMP1 (range 0.156–10 ng/mL, sensitivity 0.05 ng/mL, cat-no MBS263032), TIMP2 (range 78–5000pg/mL, sensitivity 39 pg/mL, cat-no MBS2880823), rat TβRIII/sBG ELISA (range 0.156–10 ng/mL, sensitivity 0.086 ng/mL; cat-no MBS289506, all from MyBioSource, San Diego, CA, USA), rat P-Smad3 ELISA (range 10–500 µg/mL, sensitivity 10 µg/mL; cat-no ab186038, Abcam, Cambridge, UK) and P-Smad2 ELISA (10–1000 µg/mL; Cell Signaling, cat-no 7384c, Frankfurt, Germany).

### 4.3. RT-PCR and qRT-PCR

The 93RS2 cells were grown as described above, then TGF-β1 or TGF-β2 (both 10 ng/mL final concentration) were added to the cells for 24 h and 48 h. Total RNA was isolated with the RNeasy Mini-Kit (Qiagen, Hilden, Germany) as described by the manufacturer. The Reverse Transcription-System (Promega, Mannheim, Germany) was used for cDNA synthesis as recommended. A total of 1 µg cDNA was used in a 25 µL PCR reaction with Taq DNA Polymerase (Bio&Sell, Feucht, Germany) and all components as mentioned in the protocol of the manufacturer. After an initial heating at 94 °C for 5 min 35 cycles were performed as follows: denaturation at 94 °C for 30 s, annealing at 59 °C for 30 s, and extension at 72 °C for 30 s. A final extension at 72 °C was done for 10 min. GAPDH was used as a house-keeping gene (Table 1). Primers were designed from the primer blast software (http://www.ncbi.nlm.nih.gov/tools/primer-blast) and were intron-spanning. 

For qRT-PCR, total RNA isolated from TGF-β1 or TGF-β2 (both 10 ng/mL) treated Sertoli cells as described above was subjected to DNase I treatment. Thereafter, first-strand cDNA synthesis was performed with Superscript^TM^ II reverse transcriptase (Invitrogen) following the instructions of the supplier. Real-time PCR amplification was done with iQ^TM^ SYBR Green Super-mix on the iCycler iQ System (Bio-Rad). Gene expression was measured after reaching the ct value and calculated using the ΔΔ*C*t method. GAPDH was used for normalization. 

### 4.4. Preparation of Cell Lysates for ELISA

Cell lysates were prepared as described in the Fluoroprofile supplier protocol. Briefly, 7 × 10^4^ cells/well were grown in 12-well plates for 24 h. After medium change to establish serum starvation in 1% FCS for 24 h, TIMP3, TGF-β1 or TGF-β2 were added in duplicates for the periods required. Untreated samples without the recombinant proteins were used as negative controls. The medium was aspirated and cells were then washed once with 1 mL/well ice-cold 1× PBS and lysed on ice in 300 µL/well cell lysis buffer containing 1× protease inhibitor cocktail. Lysates were collected with cell scrapers as individual samples and centrifuged at 13,000 × *g* for 10 min at 4 °C. The protein concentration in all lysates was measured using the Fluoroprofile protein quantification kit. Lysate aliquots were stored at −20 °C until further use.

### 4.5. Collection of Supernatants for ELISAs

The 1 × 10^5^ 93RS2 cells/mL were grown in 12-well plates for 24 h and serum starved in medium with 1% FCS for an additional 24 h. Cells were stimulated in duplicate wells with of TGF-β1 or TGF-β2 (both 10 ng/mL) or TIMP3 (10 nM) for 24 h and 48 h. Untreated samples without the recombinant proteins were used as negative controls. The supernatants were collected in duplicates in Eppendorf tubes with 1% protease inhibitor cocktail and stored at −20 °C until further use. The cells were washed once with 1× PBS and detached with 500 µL/well accutase for 4 min at 37 °C. Twenty µL of the single cell suspension was stained with Trypan Blue to determine the viable cell number using an automated cell counter system (TC10, Bio-Rad, Dussseldorf, Germany). 

### 4.6. TIMP1–3, TGF-β1/-β2 and TβRIII/sBG ELISA

Concentrations of TIMP1–3, TGF-β1/β2 and TβRIII/sBG in supernatants from 93RS2 cell cultures were measured by ELISA according to the manufacturer’s instructions. For the TGF-β1/β2 ELISA samples were activated by 1N HCl and 1.2N NaOH. Briefly, 100 µL supernatant/well was added to a 96-well microplate (Thermo Scientific, Schwerte, Germany) coated with capture antibody overnight at room temperature. The wells were washed three times with washing buffer followed by incubation with 100 µL detection antibody for 2 h at room temperature. After repeating the washing steps, 100 µL/well substrate solution was added for 20 min at room temperature in the dark. The reaction was stopped with 100 µL stop solution and the absorbance was determined with a microplate reader (M200, Tecan, Männedorf, Switzerland) set to 450/550 nm. Data were standardised against 1 × 10^5^ viable cells.

### 4.7. P-Smad2 ELISA

93RS2 Sertoli cells grown under standardized conditions in 12-well plates were pre-treated with TIMP3 for 2 h, followed by treatment in duplicate wells with TGF-β1 or TGF-β2 for 2 h. Untreated samples without the recombinant proteins were used as negative controls for the P-Smad3 and P-Smad3 ELISA. 

After washing with washing buffer cell lysates were prepared and equal protein amounts (50 µL) of the samples and the anti P-Smad3 antibody cocktail were added to a 96 well plate. The plate was incubated for 1 h on a plate shaker at 400 rpm, washed 3 times with 1× wash buffer provided in kit and TMB substrate was added. The absorbance of each sample was determined at 450/550 nm using an M200 microplate reader and values were normalized to the total protein content determined with the Fluoroprofile protein quantification kit4.

### 4.8. P-Smad3 ELISA

After protein quantification of the cell lysates equal protein amounts were loaded in the 96-well plate (100 µL/well) provided in the kit and incubated for 2 h at 37 °C. Then the plate was washed 4 times with 1× wash buffer. After addition of the detection antibody to each well, the plate was incubated for 1 h at 37 °C. The washing steps were repeated three times, followed by addition of HRP and TMB substrate. The absorbance was measured at 450/550 nm using an M200 microplate reader and values were normalized to the total protein content determined with the Fluoroprofile protein quantification kit. 

### 4.9. siRNA Treatment of 93RS2 Cells

The 1× 93RS2 cells were treated with siRNA for TIMP3 (100 nM; cat-no. 4390771, id s129582) or betaglycan (100 nM; cat-no. AM16708, id 50296) according to the protocols of the suppliers. As a negative control 93RS2 cells were treated with the negative control siRNA No. 2 (100 nM; cat-no. 4390846, Invitrogen). After incubating the 93RS2 cells with the siRNAs for 48 h, total RNA was prepared. Successful silencing was evaluated with RT-PCR. The 7 × 10^4^ siRNA-transfected cells were seeded in 12-well plates and stimulated with TGF-β1 or TGF-β2 (both 10 ng/mL) for 1 h. Cell lysates were collected and the P-Smad2 and P-Smad3 ELISAs performed as described.

### 4.10. BrdU Proliferation Assay

The colorimetric BrdU Assay kit (Abcam, Cambridge, UK) was used as recommended. Briefly, 2 × 10^4^ cells were seeded in a 96-well plate and allowed to grow with or without for TGF-β1 or TGF-β2 (each 10 ng/mL) for 24 h under serum free conditions. Four hours before the end of the experiment BrdU was added to the cells. After fixation the cells were incubated with an anti-BrdU antibody and afterwards with a horseradish peroxidase conjugated secondary antibody. Tetra-methylbenzidine was added as a substrate and after 30 min the reaction was stopped. Colorimetric intensity was measured at 450/550 nm with a Tecan infinite M200 ELISA plate reader.

### 4.11. Statistical Analysis

Each experiment was run in duplicate. For statistical analysis data from three independent experiments (carried out at different days) were considered (*n* = 3 × 2). Means and SEM values of all experiments were used for analysis. Comparisons of the means between more than two groups were performed by one-way analysis of variance (ANOVA) followed by Dunnett’s multiple comparison test. Student’s *t*-test was used for comparison of two groups using GraphPad prism software (Version 7.0, Graphpad Inc. La Jolla, CV, USA) *p* values ≤ 0.05 were considered significant. Significances are indicated in the figures.

## Figures and Tables

**Table 1:** List of primer sequences used for RT-PCR.

Gene (Species)	Sequence (5′→3′)	AT	Size (bp)	Acc. No.
*Betaglycan* (rat)	CTGCGAGGCAAGTTGAACAG fwd GGAGTTGAGCAGGAACACGA rev	59 °C	424	NM_0172256.1
*TIMP3* (rat)	CAATTTCGGGTACCCTGGCT fwd TGGAAGTGCGGTCTCATTCT rev	59 °C	394	NM_012886.2
*GAPDH* (rat)	GCATCTTCTTGTGCAGTGCC fwd ACTGTGGTCATGAGCCCTTC rev	59 °C	500	NM_017008.4

AT; annealing temperature; Acc. No., Accession number; fwd, forward; rev, reverse.

## Supplementary Materials

Supplementary materials can be found at https://www.mdpi.com/1422-0067/20/24/6214/s1.
