# Supplementary material for: Betaglycan (TβRIII) is a Key Factor in TGF-β2 Signaling in Prepubertal Rat Sertoli Cells
Source: Int J Mol Sci. 2019 Dec 9;20(24):6214. doi: 10.3390/ijms20246214 (PMC6941059; doi:10.3390/ijms20246214)
Supplement: Supplementary file 1 [file ijms-20-06214-s001.pdf]

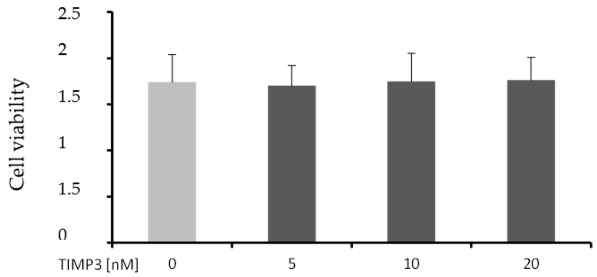

Figure S1. Effects of TIMP3 on cell viability. The 7 × 10^4 93RS2 cells/well were incubated with different doses of TIMP3 for 48 h. Cell viability was analyzed by trypan blue and showed no effects. Each bar represents the mean ± SEM of 3 independent experiments performed in duplicate. Dunnett's test was used for statistical analysis.
